# Editorial: Non-tuberculous mycobacteria and bronchiectasis

**DOI:** 10.3389/ftubr.2026.1819209

**Published:** 2026-04-01

**Authors:** Octavio M. Rivero-Lezcano, Ramiro López-Medrano, Jaime Esteban

**Affiliations:** 1 Unidad de Investigación, Complejo Asistencial Universitario de León, Gerencia Regional de Salud de Castilla y León (SACYL), León, Spain; 2 Institute of Biomedical Research of Salamanca (IBSAL), Salamanca, Spain; 3 Institute of Biomedicine (IBIOMED), University of León, León, Spain; 4 Servicio de Microbiología Clínica, Complejo Asistencial Universitario de León, Gerencia Regional de Salud de Castilla y León (SACYL), León, Spain; 5 Department of Clinical Microbiology, IIS-Fundación Jiménez Díaz, Madrid, Spain; 6 Facultad de Medicina, Universidad Autónoma de Madrid, Madrid, Spain; 7 CIBERINFEC-CIBER de Enfermedades Infecciosas, Madrid, Spain

**Keywords:** bronchiectasis, cystic fibrosis, epidemiology, granuloma, *Mycobacterium abscessus*, *Mycobacterium avium* complex, non-tuberculous mycobacteria

The epidemiological significance of non-tuberculous mycobacterial (NTM) diseases was not recognized until the early 1950s, many decades after Koch announced the discovery of *Mycobacterium tuberculosis* in 1882. It was not until the mid-1980s that clinicians described the non-classic, nodular-bronchiectatic form of NTM pulmonary infection, distinguishing it from the previously recognized classic, cavitary presentation. By the late 1980s, bronchiectasis itself was still regarded as an orphan disease. Since then, the field has rapidly evolved into one of the fastest-growing areas of interest in respiratory medicine, yet research in NTM infections continues to lag markedly behind that devoted to tuberculosis. This editorial examines the contributions included on the topic “*Non-tuberculous mycobacteria and bronchiectasis*.” This collection includes one review that explores the cause-and-effect relationship between the two conditions (Singh et al.), and a case report of an infedtion caused by the uncommon species Mycobacterium colombiense which led to the development of bronchiectasis (Tan et al.) along with three original articles. The first study examines the epidemiology of the infection and concludes that the available evidence does not support person-to-person transmission of the disease (Rubio et al.). The next article analyzes the differences in granuloma formation induced by *M. tuberculosis* and *M. avium* (Doratte et al.). The final one describes a murine model that replicates key features of cystic fibrosis, a condition closely associated with the development of bronchiectasis (Pearce et al.).

Rubio et al. investigated pathogen transmission by analyzing the cluster structure of 46 *M. avium* complex isolates obtained from patients with bronchiectasis, using whole-genome sequencing. Bronchiectasis provides a favorable environment for mycobacterial proliferation and facilitates their expulsion from the lungs through coughing. The researchers identified two clusters, each composed of two strains from different patients, isolated more than a year apart and without any evident epidemiological link, prompting the conclusion that person-to-person transmission had not occurred. If these strains share a common ancestor, the mode of transmission remains unresolved and is likely to involve indirect routes such as fomites—an area currently under active investigation. This study also offers valuable insights into the genomic diversity of *M. intracellulare* and *M. avium* species.

Among the species most frequently isolated today are *M. avium, M. intracellulare*—investigated by Rubio et al.—and *M. abscessus*. New NTM species, however, continue to be identified in clinical settings. An interesting example is the case reported by Tan et al., describing the isolation of *M. colombiense*, a member of the *M. avium* complex, from a patient with bronchiectasis. Taken together, this and other cases reported in the scientific literature suggest that bronchiectasis—initially associated primarily with *M. avium* infection—can develop in the context of infection by a wide range of mycobacterial species. The authors highlight the need for heightened surveillance for NTM in bronchiectasis patients. It remains uncertain whether any mycobacterial species—including those not previously associated with human disease—are capable of inducing bronchiectasis in a susceptible host. Notably, the genus *Mycobacterium* contains more recognized pathogenic species than any other bacterial genus (1).

Once in the lungs, if innate immunity fails to halt the multiplication of the bacilli, granuloma formation may ensue. Granulomas arising from NTM infection often appear on imaging as pulmonary nodules, a hallmark of the nodular-bronchiectatic form of the disease. It remains unclear which specific granuloma-associated factors drive cavity formation in tuberculosis, whereas NTM infections are more commonly associated with nodules and bronchiectasis. Doratt et al. address this important question by comparing tuberculous and non-tuberculous granulomas in a highly relevant primate model. Their findings show that *M. tuberculosis*-induced granulomas display a more aggressive and destructive profile, whereas those formed in response to *M. avium* exhibit signaling patterns that maintain granuloma integrity. This more stable granuloma structure may help shield the mycobacteria, promoting their persistence and the progression to chronic disease, a process in which radiologic findings may evolve from isolated nodules to established bronchiectasis (2). The evidence generated in this study may help advance ongoing efforts to understand the differing pathogenic mechanisms of the *M. tuberculosis* complex and NTM.

The genetic disease most strongly associated to bronchiectasis is cystic fibrosis caused by mutations in the gene encoding cystic fibrosis transmembrane conductance regulator (CFTR), a chlorine/bicarbonate channel that balances fluid homeostasis in epithelia. In the airways, mucus becomes dehydrated and mucociliary clearance is severely impaired, creating conditions that facilitate infection by a variety of microorganisms, including mycobacteria. A cystic fibrosis-like phenotype is reproduced in the mice by mutating the βENaC channel, which enhances Na? transport (3). Given that nearly all patients with cystic fibrosis eventually develop bronchiectasis, this model was used by Pearce et al. to examine the role of *M. abscessus*, a NTM species frequently isolated in this population. The results showed that the mice were able to clear the mycobacteria, leading to the conclusion that this model is not suitable for studying chronic infection. Nevertheless, a decline in lung function was observed, suggesting that the model may still be useful for investigating the early stages of bronchiectasis development—a possibility that warrants further study.

The review by Singh et al. may serve as a compendium of the subject matter covered in this Research Topic. The authors describe the vicious cycle that arises between NTM infection and bronchiectasis. NTM infection fuels persistent inflammation and tissue damage through cytokine release and granulomatous responses, leading to further architectural distortion of the airways and progression of bronchiectasis. In turn, the structural abnormalities and impaired mucociliary clearance characteristic of bronchiectasis increase susceptibility to NTM acquisition, perpetuating the cycle. The authors also emphasize other key aspects of the disease, such as the diagnostic challenges faced in high-burden countries, where smear microscopy cannot distinguish *M. tuberculosis* from NTM, making molecular methods essential, as well as the difficulty of differentiating true infection from mere colonization. According to international guidelines, confirmation of NTM pulmonary disease requires a combination of clinical symptoms, radiological abnormalities, and microbiological evidence, including at least two positive sputum cultures or one positive bronchoscopy sample. The importance of biofilm formation is also stressed, as it promotes microbial persistence and facilitates horizontal gene transfer, thereby potentially increasing antimicrobial resistance and complicating treatment.

## Concluding remarks

The association between NTM infection and bronchiectasis affects an ever-growing number of patients, yet many aspects of their interplay remain poorly understood. The simultaneous rise in the incidence of both conditions is unlikely to be coincidental and may instead reflect a close and biologically meaningful connection. Moreover, the proportion of infections that progress to bronchiectasis and the proportion of pre-existing structural airway abnormalities that predispose to infection are unlikely to be equivalent. In each scenario, the therapeutic priorities would differ, creating opportunities for more tailored and personalized treatment strategies.
